# *Blastocystis* specific serum immunoglobulin in patients with irritable bowel syndrome (IBS) versus healthy controls

**DOI:** 10.1186/s13071-015-1069-x

**Published:** 2015-09-15

**Authors:** Robyn Nagel, Rebecca J. Traub, Marcella M S Kwan, Helle Bielefeldt-Ohmann

**Affiliations:** School of Veterinary Science, The University of Queensland, Gatton Campus, Gatton, QLD 4343 Australia; Faculty of Veterinary and Agricultural Sciences, University of Melbourne, Parkville, VIC 3052 Australia; Rural Clinical School, School of Medicine, The University of Queensland, Toowoomba, 4350 Australia; Australian Infectious Diseases Research Centre, The University of Queensland, St. Lucia, QLD 4072 Australia; Toowoomba Gastroenterology Clinic, Suite 105 Medici Medical Centre, 15 Scott St, Toowoomba, QLD 4350 Australia

**Keywords:** *Blastocystis*, Irritable bowel syndrome, Immunoglobulin A, Western blotting, proteases

## Abstract

**Background:**

*Blastocystis* species are common enteric human parasites and carriage has been linked to Irritable Bowel Syndrome (IBS), particularly diarrhoea-predominant IBS. The spectrum of immune reactivity to *Blastocystis* proteins has been reported previously in symptomatic patients. We investigated differences in serum immunoglobulin profiles between patients with IBS, both positive and negative for *Blastocystis* carriage, and healthy controls (HC).

**Methods:**

Forty diarrhoea-predominant IBS patients (26 patients positive for *Blastocystis* sp*.,* 14 negative patients) and forty HC (24 positive, 16 *Blastocystis-*negative) were enrolled. Age, gender, ethnicity and serum immunoglobulin A (IgA) levels were recorded and faecal specimens were analysed using smear, culture and polymerase chain reaction amplification of ribosomal DNA. Sera were tested in Western blots and the reactivities compared to known targets using monoclonal antibodies Blastofluor® (*Blastocystis* specific antibody), MAb1D5 (cytopathicto *Blastocystis* cells), anti-promatrix metalloprotease-9 (anti-MMP-9) and SDS-PAGE zymograms.

**Results:**

Levels of serum IgA were significantly lower in *Blastocystis* carriers (*p* < 0.001) but had no relationship to symptoms. Western blots demonstrated serum IgG antibodies specific for *Blastocystis* proteins of 17,27,37,50,60-65, 75–90, 95–105 and 150 kDa MW. Reactivity to the 27, 50 and 75-95 kDa proteins were found more frequently in the IBS group compared to the HC’s (*p* < 0.001) and correlation was greater for *Blastocystis*-positive IBS patients (*p* < 0.001) than for negative IBS patients (*p* < 0.05). MAb1D5 reacted with proteins of 27 and 100 kDa, and anti-MMP-9 with 27, 50 and 75-100 kDa proteins. Bands were seen in zymograms around 100 kDa.

**Conclusions:**

Low serum IgA levels are associated with *Blastocystis* carriage. All IBS patients were more likely to demonstrate reactivity with *Blastocystis* proteins of 27 kDa (likely a cysteine protease), 50 and 75-95 kDa MW compared to HC. The presence of antibodies to these *Blastocystis* proteins in some *Blastocystis*-negative subjects suggests either prior exposure to *Blastocystis* organisms or antibody cross reactivities. The anti-proMMP-9 reaction at 50 and 75–100 kDa and the zymogram result suggest that metalloproteases may be important *Blastocystis* antigens.

**Trial registration:**

Australian and New Zealand Clinical Trials registry ACTRN: 12611000918921

**Electronic supplementary material:**

The online version of this article (doi:10.1186/s13071-015-1069-x) contains supplementary material, which is available to authorized users.

## Background

Irritable bowel syndrome (IBS) is a common clinical condition affecting up to 10 % of the global population and characterised by abdominal pain, bloating and disturbance of bowel habit [1]. The underlying cause of IBS is not known although differences in gut motility, the enteric immune and nervous system, intestinal permeability, systemic cytokine production, faecal protease excretion, faecal microflora and psychological profiles have been reported in this condition [2]. The disease is defined by a clinical symptom complex description (Rome III) [3] that has a sensitivity of 70 % and a specificity of 80 % in differentiating IBS patients from patients with other gastrointestinal diseases [4]. This clinical definition is the gold standard, but assessment can be subjective. Reliable, clinically applicable IBS biomarkers, particularly if they direct therapeutic choices, would be useful. Serum biomarkers for IBS have been investigated in two previous studies [5, 6] and both were less accurate than using the Rome III criteria. Post-infectious IBS is known to comprise 10 % of the total cases of IBS and this has stimulated interest in the faecal microbiota as a possible contributing cause of IBS [7]. The carriage of the enteric organism *Blastocystis* has been reported to be three times higher in patients with diarrhoea predominant IBS (D-IBS) compared to healthy controls [8], making it an organism of interest in IBS.

*Blastocystis* sp. are the most common parasites found in human stool [9]. Sub-typing of the18S ribosomal DNA(rDNA) has identified 17 different subtypes (ST’s) and nine have been identified in humans [10, 11]. Carriage of *Blastocystis* sp. is increased in patients with various types of immunosuppression [9] and in patients with irritable bowel syndrome [8]. Nevertheless, many carriers are healthy and a definite association between carriage and illness has not been proven in epidemiological studies [12].

*Blastocystis* sp. reside in the intestinal lumen, establishing in the ileum and caecum adherent to the outer layer of mucus [13, 14], with only rare reports of mucosal invasion. Antibodies specific for *Blastocystis* antigens have been demonstrated in the faeces and the serum of carriers [15–19] and antibody titres have been reported to be higher with length and severity of infection [15]. These antibodies have been described in all immunoglobulin classes. IgA antibodies specific for*Blastocystis* sp. have been shown to be present in pig faeces [19] and in both faeces and serum of infected humans [20]. Our previous pilot study demonstrated lower serum IgA levels in IBS patients positive for *Blastocystis* carriage than in other patient groups or healthy controls [21].

*Blastocystis* specific immunoglobulin G (IgG) antibodies have also been detected in serum [15, 20, 22] and faeces of *Blastocystis* infected humans [20], with lower titres in the latter compartment. Using SDS-PAGE and Western blot analysis, serum IgG has been reported to react with *Blastocystis*-specific proteins of 12 kDa [16], 29 kDa, 50 kDa and 118 kDa molecular weights (MWs) [17]. Antibody reactivity to a 29 kDa MW *Blastocystis* protein has been reported to be more common in the serum of symptomatic patients compared to asymptomatic individuals [18], and subsequent protein sequencing of a 30 kDa protein showed it to possess 50 % homology with known cysteine protease (legumain type) peptide sequences [23]. A monoclonal IgM antibody 1D5 (MAb1D5), known to be cytopathic to *Blastocystis* organisms [24] as well as a human legumain antibody both bind to this 30 kDa *Blastocystis* antigen in Western blots.

Protease secretion is a recognised virulence mechanism for parasites [25] facilitating tissue/cell invasion, protein activation and immunoevasion. These molecules are also highly immunogenic. Analysis of the genome of *Blastocystis* ST7 has led to the predictions that the parasite would be able to produce all major classes of proteases, including serine proteases, metalloproteases and perhaps as many as 20 different cysteine proteases [26, 27]. Notably *Blastocystis* proteases have been shown to cleave human secretory IgA [28] and induce production of the pro-inflammatory cytokine interleukin-8 (IL-8) by enterocytes in an *in vitro* model system [29].

Our aim was to explore the clinical relevance of *Blastocystis* sp. in patients with diarrhoea predominant IBS by assessing serum antibody reactivities specific for *Blastocystis*. In this study we compared the serum IgA levels in IBS patients, either positive (IBS-P) or negative (IBS-N) for *Blastocystis* carriage, and healthy controls (positive (HC-P) and negative (HC-N) for *Blastocystis*). IgG serological responses to specific *Blastocystis* antigens were examined in all these subgroups using Western blotting techniques. Identification of specific *Blastocystis* antigens was attempted by probing the Western blots with *Blastocystis* specific antibody (Blastofluor®, Antibodies Inc), MAb1D5 and pro-matrix metalloprotease 9 (anti-MMP-9) antibody.

## Methods

### Study outline

Forty patients presenting with diarrhoea-predominant IBS to the Toowoomba Gastroenterology Clinic and forty healthy volunteers were enrolled in the study and signed written consent forms. The study was approved by the University of Queensland Medical Research Ethics Committee and was part of a clinical trial that is registered with the Australian and New Zealand Clinical Trials registry (http://www.ANZCTR.org.au) ACTRN: 12611000918921. Single baseline faecal and serum samples were collected from all participants. Total serum IgA levels were measured in all participants and faecal and serum specimens underwent further microbiological, molecular and Western blot analysis.

### Ethical approval

The study was approved by the University of Queensland Medical Research Ethics Committee (No 2011000454).

#### Inclusion protocol

Adult patients presenting with chronic diarrhoea and abdominal pain from 1/8/11 to 20/02/14 were assessed clinically. Baseline blood tests, including full blood count, serum calcium, thyroid function tests, serum IgA and coeliac antibody tests were performed. Other pathogens known to cause chronic diarrhoea such as *Salmonella* sp, *Shigella* sp, *Vibrio*sp., *Campylobacter* sp, *Aeromonas* sp),*Giardia duodenalis*, *Clostridium difficule* and *Dientamoebahistolytica* were excluded with faecal microscopy , culture and PCR testing performed by a commercial pathology laboratory. All patients proceeded to an upper and lower endoscopy that included gastric antral, duodenal, ileal and colonic biopsies. Forty eligible symptomatic patients who had no other cause for symptoms and who fulfilled the Rome criteria [30] for diarrhoea predominant IBS were enrolled in the study. Of these, 26 patients were positive for *Blastocystis* (IBS-P) and 14 were negative (IBS-N). Forty healthy volunteers working at the University of Queensland and with no gastrointestinal symptoms in the preceding 12 months were tested for *Blastocystis* sp. infection, 24 were positive (HC-P) and 16 were negative (HC-N). Details of age, gender and ethnicity of participants were recorded.

#### Exclusion protocol

Only non-pregnant adults between 18 and 75 years of age were recruited for the study. Patients with significant systemic diseases or co-morbidities were excluded.

### Diagnosis of *Blastocytis* infection

All samples were run in parallel for the presence of *Blastocystis* sp. using a simple unstained wet faecal smear, xenic *in vitro* culture (XIVC) and PCR (confirmed as *Blastocystis* sp. using DNA sequencing). A study participant was considered to be positive if any one of the tests was positive as described previously [21].

### Serum Immunoglobulin A

Serum samples were stored at −20 °C prior to analysis at Sullivan and Nicolaides Pathology Service, Brisbane, Queensland using a Siemens BNII Nephelometer (Siemens, Munich, Germany) and Siemens reagents.

### Western blotting

#### Antigen preparation

An axenic strain of *Blastocystis* sp. ST4 (WR1) was cultured anaerobically in pre-reduced Iscove’s Modified Dulbecco’s medium (IMDM) (Sigma-Aldrich, St Louis, USA: 13390) enriched with 10 % heat-inactivated horse serum (Gibco: 26050088). WR1 was originally obtained from the stool of Wistar rats in Singapore but has been in continuous axenic culture for more than seventeen years. *Blastocystis* antigen was prepared using the proteinase inhibitor Complete Lysis-B (2x), EDTA-free kit (Roche, Mannheim, Germany:04719948001) and stored at −20 °C until required.

#### SDS-PAGE protocol

Approximately 300 ng of *Blastocystis* antigen was mixed with equal volumes of loading buffer (Laemmli sample buffer mixed with 2 M 2-mercaptoethanol in a ratio of 19:1vol:vol (Bio- Rad, California, USA)) and loaded into the large well of a preparative 4-15 % Tris–HCl Ready Gel (Bio-Rad: 161–1140). A separate single protein standard ladder well in the gel was loaded with 15uL of Precision Plus Protein Western C Standard (Bio-Rad: 161–0385). The gel was electrophoresed in M Tris/Glycine/SDS buffer (Bio-Rad: 161–0732) at 100 V for 40–60 min at room temperature and then rinsed three times with deionised water. The proteins were transferred to a nitrocellulose membrane using 1 M Tris/Glycine/20 % methanol buffer electrophoresed at 100 V for 60 min at 4 °C. The membrane was rinsed three times with 0.1 % 1Mphosphate buffered saline (PBS)/Tween 20 (PBST) (Sigma Aldrich, St Louis, USA: P5927) solution and then blocked in 100 ml of blocking solution (0.1 % PBST, 4g skim milk and1g bovine serum albumin per 100 ml (Sigma Aldrich: A2153) for 60 min at room temperature. The membrane was cut into strips and stored at −20 °C till required.

#### Immunolabeling protocol

Membrane strips were incubated with primary antibody (patient sera or MAbs) diluted in 1 mL of Signal Boost™Immunoreaction Enhancer Kit (Calbiochem, Darstadt, Germany: 407207) Solution One for 24 h on a rocker platform at 4 °C before washing consecutively three times for 15 min in PBST. The strips were then incubated with secondary antibody diluted in Signal Boost Solution Two at room temperature for 1 h on a rocker platform and the washing steps repeated.

The patient sera were diluted 1:10 and the secondary antibody was a 1: 2000 dilution of horseradish peroxidase (HRP)-conjugated goat anti-human IgG Ab (Sigma Aldrich:A8786).

An additional four antibodies were tested for reactivity with the *Blastocystis* antigens:(i) a mouse derived *Blastocystis*-cytopathic monoclonal IgM antibody, MAb1D5 (purified Mab against surface legumain of Blastocystis ST7, IgM, 16 μg/μL) [27, 31, 32], (ii) a control unrelated mouse derived monoclonal IgM antibody, MAb5, (iii) a rabbit polyclonal anti-*Blastocystis* ST3 antibody (Blastofluor®) [33],and (iv) a rabbit polyclonal anti-human matrix metalloproteinase 9 (Anti-MMP-9) (Calbiochem: 444236). These primary antibodies were diluted 1:100, and the secondary antibodies1:1000, using HRP-conjugated-goat anti-mouse IgM (Sigma, St Louis, USA: Cat no: A8786) and HRP-conjugated anti-rabbit polyclonal Ab, respectively.

All blots were run with a protein standard ladder and a negative control. Negative controls were prepared according to the protocol above except exclusion of serum/primary antibody in step one followed by incubation with the secondary antibody.

The antibody binding was visualized using Clarity Western ECL Substrate (Bio Rad, California USA) and a Bio Rad Chemi-Doc^TM^ XRS Gel Documentation system. The brightness and contrast was adjusted over entire images to optimise the image but no differential image manipulation was used on separate lanes.

### Zymograms

The *Blastocystis* antigen was prepared as per the Western blotting protocol and combined with Zymogen buffer (Bio-Rad: 161–0764) in a vol:vol ratio of 1:1. Approximately 50 ng of buffered *Blastocystis* antigen was loaded into each well of a 10 % gelatin Ready Gel® Zymogram Gel (Bio-Rad: 161–1167) and the gel was electrophoresed in 1 M Tris/Glycine/SDS buffer (Bio-Rad: 161–0732) at 90 V for 40–60 min at room temperature. The gel was cut in half and both gels subjected to renaturation and development steps. The gels were incubated in 100 mL of Zymogen Renaturation buffer (Bio-Rad: 161–0765) for 1 h at room temperature on a rocker platform and then incubated in 100 mL of Zymogen Development buffer (Bio-Rad: 161–0766) for 24 h at 37 °C without or with protease inhibitor, 1 mM EDTA (ethylenediaminetetraacetic acid), added to the two solutions for one gel. The buffer was decanted and the gels were stained with 100 mL Aqua® stain (Bulldog Bio, Portsmouth, UK) for 15 min on a rocker platform and rinsed with deionised water.

### Statistical analysis

Statistical analysis was carried out using SPSS v.22 [34]. The level of IgA was transformed using natural logarithm to get a normal distribution. A one-way Analysis of Covariance (ANCOVA) was used to compare the level of IgA between the four groups, adjusting for potential confounders, such as seasonality [35]. Pearson’s chi-square/ Fisher exact test were used to assess the significance of association between subjects with IBS and healthy controls with the presence of serum IgG antibodies detected against *Blastocystis* proteins of various sizes.

## Results

### Study subjects

The eighty study participants comprised forty symptomatic IBS patients (26 positive and 14 negative for *Blastocystis* carriage) and forty asymptomatic healthy controls (24 positive and 16 negative for *Blastocystis*). The average participants’ age was 45.2 ± 14.4 years (range 16–75 years). There were no significant between-group differences in terms of age (F(3,76) = 0.81, *p* = 0.49) and gender distribution (λ^2^ = 4.37, *p* = 0.22). However, there were significantly more Caucasians compared to non-Caucasians in the IBS-P, IBS-N and HC-P groups (λ^2^ = 16.83, *p* = 0.001). The *Blastocystis* sp. subtypes detected were ST3 (34 %), ST4 (26 %), ST1 (16 %), ST2 and ST7 (8 %), ST8 (6 %) and ST5 (2 %). Characteristics of the 80 subjects and their *Blastocystis* subtypes are given in Tables 1 and 2.

**Table 1:** Demographic and epidemiological characteristic of subgroups

	IBS-P	IBS-N	HC-P	HC-N
	(*n* = 26)	(*n* = 14)	(*n* = 24)	(*n* = 16)
Age (years; mean ± SD)	44.5 ± 15.2	44.5 ± 14.2	48.7 ± 13.2	41.7 ± 15.0
Female (*n*, %)	19	11	12	11
Caucasian (*n*, %)	26 (100)	13 (93)	21 (88)	9 (56)
Season recruited (*n*)				
Spring	2	3	19	0
Summer	8	5	4	1
Autumn	11	4	0	7
Winter	5	2	1	8
*Blastocystis* subtype distribution				
ST1	2		6	
ST2	1		3	
ST3	9		8	
ST4	9		4	
ST5	0		1	
ST6	0		0	
ST7	3		1	
ST8	2		1	
Serum IgA^a^ (g/L; mean ± SD)	1.63 ± 0.95	2.19 ± 1.01	1.54 ± 0.76	3.06 ± 1.61

*SD*: standard deviation

*IBS-P/N*: Irritable bowel syndrome subjects positive/negative for Blastocystis

*HC-P/N*: Healthy control subjects positive/negative for Blastocystis

*ST*: subtype; *IgA*: Immunoglobulin A

^a^ unadjusted mean; results IgA of different subtypes in IBS-P & HC-P groups were pooled for analysis purpose

**Table 2:** Serum antibody reactions to *Blastocystis* antigens detected by Western immunoblotting in all subjects infected with different subtypes of *Blastocystis*

	

### Serum IgA levels

Serum IgA levels were higher in male (2.36 ± 1.36 g/L) compared to female participants (1.79 ± 1.09 g/L) (t = 4.15, *p* < 0.05). Levels of IgA were not found to be different between Caucasian and Asian participants, nor across the different *Blastocystis* subtypes (both *p* > 0.05).

The average level of serum IgA was lowest in the HC-P group, followed by IBS-P, IBS-N and HC-N (Table 1). Participants who tested *Blastocystis-*positive (combined IBS-P and HC-P subgroups) had a significantly lower serum IgA level (1.59 ± 0.54 g/L) than their *Blastocystis-* negative counterpart (combined IBS-N and HC-N) subgroups (2.65 ± 1.41 g/L) (t(78) = 4.06, *p* < 0.001). Planned contrasts revealed that participants in the HC-N group had significantly higher serum IgA levels compared to those in the IBS-P group, t(75) = −3.31, *p* = 0.001, and HC-P group, t(75) = −4.30, *p* < 0.001. Pairwise comparisons showed that IgA levels in IBS-N participants were higher than those in the HC-P group (*p* < 0.01).

There was a significant difference in IgA levels between the four groups after controlling for the potential confounding effect of seasonality and gender, F(3,73) = 9.05, *p* < 0.001. The gender covariate was significantly related to the level of IgA, (F(1,73) = 7.93, *p* < 0.01), whereas seasonality, was not (F(1,75) = 3.42, *p* = 0.07).

### *Blastocystis*-specific serum IgG antibody bands

**Fig. 1:**
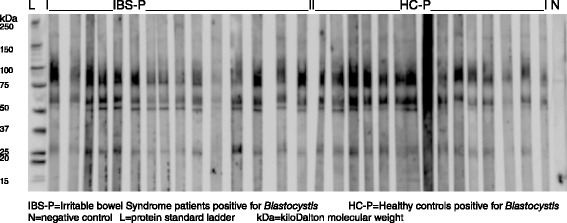
Serum antibodies from *Blastocystis* positive clinical subgroups IBS-P and HC-P reacting with *Blastocystis* proteins in Western blot

**Fig. 2:**
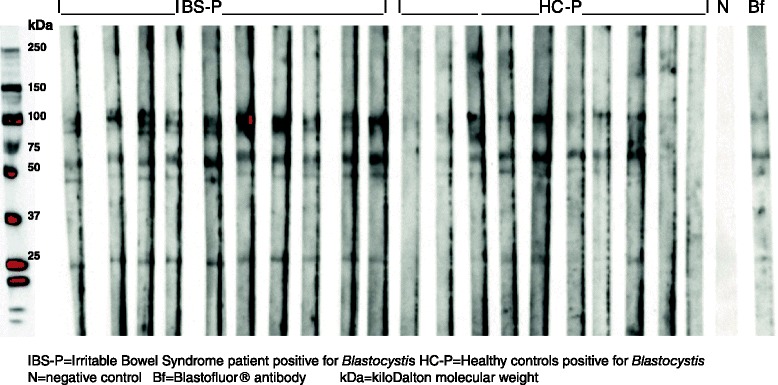
Serum antibodies from *Blastocystis*–positive clinical subgroups IBS-P and HC-P, and Blastofluor® Ab reacting with *Blastocystis* proteins in Western blot

**Fig. 3:**
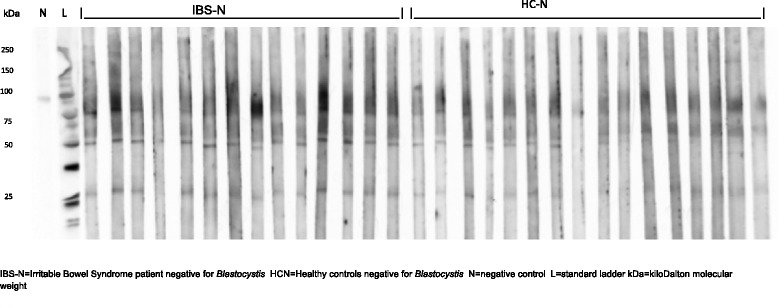
Serum antibodies from *Blastocystis* negative clinical subgroups IBS-N and HC-N reacting with *Blastocystis* proteins in Western blot

Western blots were employed to assess the serum IgG response to *Blastocystis* proteins in the four clinical groups and the analysis of these results is shown in Tables 2, 3 and 4. Reactivity was seen to *Blastocystis* proteins of approximately 27, 50, 60–65, 75–90, 95–105 and 150kDaMW [36] in all groups of patients (Figs. 1, 2, 3). These results were examined to assess whether serum reactivity to *Blastocystis* proteins of different sizes was associated with carriage of a particular subtype of *Blastocystis* sp., or either the presence of *Blastocystis* sp. orIBS symptoms. If Blastocystis sp. were to prove to be a cause of IBS then it is possible that IBS-N patients may have had *Blastocystis* infection in the relatively recent past and the subgroups with IBS were analysed separately in order to reduce any contribution from pre-existing antibodies (Table 4).

**Table 3:** Serum antibody reactions to *Blastocystis* antigens detected by Western immunoblotting in different clinical groups

Protein band at molecular weight	Number of subjects with presence of protein bands (*n*, %)			
	IBS-P (*n* = 26)	IBS-N (*n* = 14)	HC-P (*n* = 24)	HC-N (*n* = 16)
17 kDa	1 (4)	0 (0)	5 (21)	0 (0)
27 kDa	25 (96)	12 (86)	17 (71)	7 (44)
37 kDa	3 (12)	0 (0)	3 (13)	0 (0)
50 kDa	26 (100)	13 (93)	16 (67)	8 (50)
60-65 kDa	26 (100)	12 (86)	19 (80)	13 (81)
75-90 kDa	25 (96)	13 (93)	14 (58)	9 (56)
95-105 kDa	14 (54)	3 (21)	14 (58)	2 (13)
150 kDa	5 (19)	1 (7)	16 (67)	1 (6)

*IBS-P/N*: Irritable bowel syndrome subjects positive/negative for Blastocystis

*HC-P/N*: Healthy control subjects positive/negative for Blastocystis

*n*: number kDa: kiloDalton

**Table 4:** Comparison of presence of *Blastocystis* and/or gastrointestinal symptoms to specific sized antibody bands directed against *Blastocystis* proteins using Western immunoblotting

		Presence of antibody to *Blastocystis* protein at band MW (n, %)															
Gp1	Gp2	17 kDa		27 kDa		37 kDa		50 kDa		60-65 kDa		75-90 kDa		95-105 kDa		150 kDa	
		Gp1	Gp2	Gp1	Gp2	Gp1	Gp2	Gp1	Gp2	Gp1	Gp2	Gp1	Gp2	Gp1	Gp2	Gp1	Gp2
Blastocystis presence																	
IBS-P & HC-P (*n* = 50)	IBS-N & HC-N (*n* = 30)	6 (12)	0 (0)*	42 (84)	19 (63)*	6 (12)	0 (0)*	42 (84)	21 (70)	45 (90)	25 (83)	39 (78)	22 (73)	28 (56)	5 (17)***	21 (42)	2 (7)***
IBS-P & HC-P (*n* = 50)	HC-N (*n* = 16)	6 (12)	0 (0)	42 (84)	7 (44)***	6 (12)	0 (0)	42 (84)	8 (50)**	45 (90)	13 (81)	39 (78)	9 (56)	28 (56)	2 (13)**	21 (42)	1 (6)**
IBS-P (*n* = 26)	IBS-N (*n* = 14)	1 (4)	0 (0)	25 (96)	12 (86)	3 (12)	0 (0)	26 (100)	13 (93)	26 (100)	12 (86)*	25 (96)	13 (93)	14 (54)	3 (21)*	5 (19)	1 (7)
HC-P (*n* = 24)	HC-N (*n* = 16)	5 (21)	0 (0)^	17 (71)	7 (44)	3 (13)	0 (0)	16 (67)	8 (50)	19 (79)	13 (81)	14 (58)	9 (56)	14 (58)	2 (13)**	16 (67)	1 (6)***
GIT symptoms																	
IBS-P & IBS-N (*n* = 40)	HC-P & HC-N (*n* = 40)	1 [56]	5 (13)	37 (93)	24 (60)***	3 (8)	3 (8)	39 (98)	24 (60)***	38 (95)	32 (80)*	38 (95)	23 (58)***	17 (43)	16 (40)	6 (15)	17 (43)**
IBS-P (*n* = 26)	HC-P & HC-N (*n* = 40)	1 (4)	5 (13)	25 (96)	24 (60)***	3 (12)	3 (8)	26 (100)	24 (60)***	26 (100)	32 (80)*	25 (96)	23 (58)***	14 (54)	16 (40)	5 (19)	17 (43)*
IBS-P (*n* = 26)	HC-P (*n* = 24)	1 (4)	5 (21)	25 (96)	17 (71)*	3 (12)	3 (13)	26 (100)	16 (67)***	26 (100)	19 (80)*	25 (96)	14 (58)***	14 (54)	14 (58)	5 (19)	16 (67)***
IBS-P (*n* = 26)	HC-N (*n* = 16)	1 (4)	0 (0)	25 (96)	7 (44)***	3 (12)	0 (0)	26 (100)	8 (50)***	26 (100)	13 (81)*	25 (96)	9 (56)***	14 (54)	2 (13)**	5 (19)	1 (6)
IBS-N (*n* = 14)	HC-P & HC-N (*n* = 40)	0 (0)	5 (13)	12 (86)	24 (60)	0 (0)	3 (8)	13 (93)	24 (60)*	12 (86)	32 (80)	13 (93)	23 (58)*	3 (21)	16 (40)	1 (7)	17 (43)*
IBS-N (*n* = 14)	HC-N (*n* = 16)	0 (0)	0 (0)	12 (86)	7 (44)*	0 (0)	0 (0)	13 (93)	8 (50)*	12 (86)	13 (81)	13 (93)	9 (56)*	3 (21)	2 (13)	1 (7)	1 (6)

Gp: group of study subjects; n: number of subjects; kDa: kiloDalton; MW: molecular weight

IBS-P/N: Irritable bowel syndrome subjects positive/negative for Blastocystis

HC-P/N: healthy control subjects positive/negative for Blastocystis

*p ≤ 0.05; **p ≤ 0.01; ***p ≤ 0.001

The numbers of *Blastocystis*-positive subjects in each subtype group were small but serum reactivity to all the protein bands was present in every subtype with the exception of 17 kDa, 37 kDa and 150 kDa bands (Table 2). No significant associations were found between *Blastocystis* sp. subtypes and reactivity to particular protein bands based on Fisher’s exact test results (all *p* > 0.05).

Reactivity with proteins of approximately 17 and 37 kDa MW were observed in 7.5 % of total participants and these were only present in those subjects who tested positive for the presence of *Blastocystis* sp. Proportions of participants with reactivity for proteins of 95-105 kDa and 150 kDa MW, were significantly lower in the combined *Blastocystis*-negative group(λ^2^ = 11.97, *p* ≤ 0.001 and λ^2^ = 11.43, *p* ≤ 0.001 respectively) (Table 3). The association of antibody-reactivity to the 95–105 kDa protein(s) with *Blastocystis* carriage was statistically significant in the intra-subgroup comparisons in both HC and IBS groups (*p* ≤ 0.05) (Table 4).

Reactivity to the 27, 50 and 75–90 kDa MW bands were detected in descending order of frequency in the IBS-P group (96 %, 100 %, 96 %), the IBS-N group (86 %, 93 %, 93 %), the HC-P (71 %, 67 %, 58 %) and the HC-N (44 %, 50 %, 56 %) groups (Table 3). Reactivity to each of these three proteins was found to be significantly associated with IBS symptoms, regardless of *Blastocystis* status (combined IBS-P + IBS-N(*p* ≤ 0.001), IBS-P (*p* ≤ 0.001) or IBS-N(*p* < 0.05), respectively, versus HC: Table 4).

### Further Analysis of the *Blastocystis* Antigens

The Blastofluor® antibody was tested in parallel with IBS-P and HC-P sera on a Western blot and showed reactivity with all the protein bands noted in the study subgroups, namely 17, 27, 37, 50, 60–65, 75–90, 95–105, 150 and 250 kDa MW (Fig. 2).

**Fig. 4:**
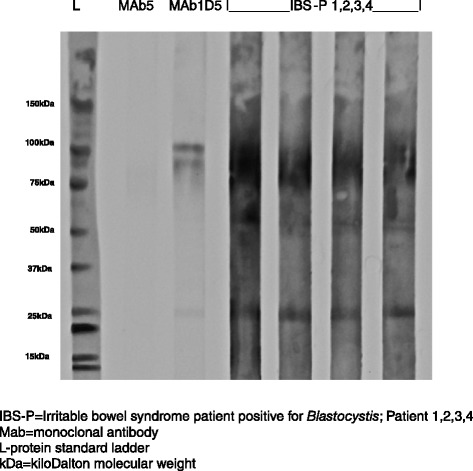
Serum antibodies from clinical subgroup IBS-P and monoclonal antibodies MAb1D5 and MAb5 reacting with *Blastocystis* proteins in Western blot

**Fig. 5:**
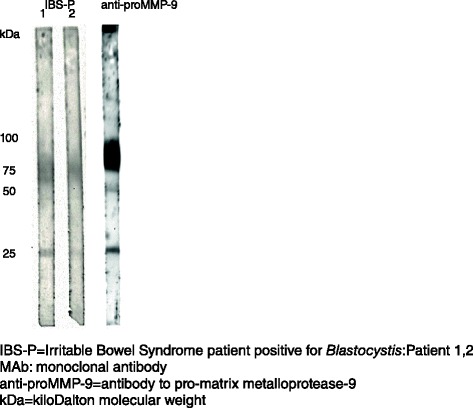
Serum antibodies in IBS-P clinical subgroup and MAb anti-proMMP-9 reacting with *Blastocystis* proteins in Western blot

The MAb1D5 and MAb5 monoclonal antibodies were tested in parallel with four sera derived from BSP patients on a Western blot. The MAb1D5 reacted strongly with two protein bands of approximately 100 kDa MW and less strongly with a27kDa protein. The MAb5 gaveno reactivity on the blot (Fig. 4). The anti-MMP-9 antibodies were tested in parallel with two BSP sera on a Western blot and the anti-MMP-9 antibodies reacted with proteins of 27, 50 and 75–100 kDa MW (Fig. 5).

The MAb1D5, MAb5, anti-MMP-9 were tested in parallel on a Western blot, using a 7.5 % gel in an attempt to separate out the bands around 75-100 kDa MW, and this demonstrated that the MAb1D5 antibody reacted with a protein closer to 100 kDa MW compared to the anti-MMP-9 that reacted with a protein of approximately 80 kDa (Additional file 1: Figure S1). Both antibodies reacted with a protein of approximately 25 kDa MW.

A zymogram was run with and without EDTA (metalloprotease inhibitor). In the absence of EDTA two distinct bands above and below the 100 kDa protein marker (approximately 95 and 105 kDa, respectively) were apparent. Addition of EDTA to the zymogram abolished these two bands (Additional file 2: Figure S2).

## Discussion

Serum IgA levels were found to be significantly lower in subjects that were positive for *Blastocystis* sp. even after adjusting for the confounding variables of gender, age, season and Asian ethnicity that have been reported to influence serum IgA levels [35, 37]. The level of IgA correlated with carriage but not symptoms suggesting that a low serum IgA may permit *Blastocystis* sp. to establish in the gut lumen but may not influence pathogenicity. The association of *Blastocystis* carriage and low serum IgA would be consistent with the fact that IgA is the major immunoglobulin class involved in mucosal defence [38] and is known to prevent adherence of pathogens to the gut luminal surface, bind toxins and inhibit antigen absorption. IgA production increases after three months of age in parallel with the change from a sterile gut to one with a complex commensal intestinal microbiota [39]. Secretory IgA can be effective against pathogens as an innate non-specific local response or develop into an adaptive specific systemic response after presentation of luminal antigen to gut associated lymphoid tissue (GALT) and subsequent generation of IgA-producing plasma cells in the intestinal mucosa. The complex relationship of IgA to the regulation or monitoring of the intestinal microbiota is not understood. Proteases derived from *Blastocystis* cell lysates have been shown to be capable of degrading secretory IgA [28].

Low serum levels of IgA may be due to a number of causes including partial or complete selective IgA deficiency [40] and likely influence host and microbial interactions. IgA deficiency is associated with an increased risk of giardiasis (caused by another non-invasive luminal parasite) as well as respiratory infections and allergic and autoimmune diseases [38]. Patients with autoimmune diseases and selective IgA deficiency more commonly exhibit the major histocompatibility complex (MHC) haplotype 8.1 and other non-MHC gene defects have been reported [41].

IgG serum antibodies from many study participants in all subgroups reacted with proteins of approximately 27, 50, 60–65, 75–90, 95–105, and 150 kDa MW in the Western blots using axenic WR1 (ST4) as antigen source. Although differences in the quantity or affinity of the antibodies were suggested by the intensity of the signals, no attempt was made to apply quantitation by densitometry. The rabbit-derived Blastofluor® antibody, raised in rabbits primed with axenic ST3, showed a very similar reactivity pattern to proteins of 10, 17, 27, 37, 50, 75, 100 KDa MW when tested in parallel with human sera (Fig. 2), suggesting that these particular *Blastocystis* antigens are common to both subtypes 3 and 4. Whilst some researchers have described different SDS-PAGE protein patterns in different *Blastocystis* organisms [42, 43] others have found little difference in the protein sizes between different *Blastocystis* subtypes [31, 44].

Over 40 % of healthy asymptomatic patients negative for *Blastocystis* sp. reacted to *Blastocystis* antigens at 27, 50, 60–65 and 75-90 kDa, respectively (Table 3), suggesting that positive reactions with these proteins occurred either due to past exposure to *Blastocystis* sp. or as a result of a cross reactivity with the *Blastocystis* antigens. Only the antibody band sizes 95–105 and 150kDA were significantly more common in those with current carriage of *Blastocystis* sp., occurring in approximately 60 % of these individuals.

However, IBS groups, either positive or negative for *Blastocystis* carriage*,* when compared to HC displayed a different pattern with significantly higher number of reactions to 27, 50 and 75-95kDaMW proteins. Overall these proteins were present in approximately 86-100 % of IBS patients compared to 44-71 % of HC. Although presence of these proteins was greatest in the *Blastocystis*-positive IBS group, it was still present in the IBS group negative for current carriage of *Blastocystis* sp*..* This finding again suggests the positive reactions in the IBS-N group were either due to non-specific antibody cross-reactions or due to residual antibody from past exposure to *Blastocystis* organisms. The gut epithelial permeability is increased in IBS [7, 45] and it is probable that IBS patients have greater exposure to intraluminal antigens. Although the number of subjects in our clinical subgroups was relatively small our findings suggest that exploration of the nature of these 27, 50, 75-95 kDa MW proteins may be useful in identifying biomarkers for IBS (current biomarkers are not able to reliably exclude organic disease [4]), identifying patients who are symptomatic due to *Blastocystis* infection and informing development of effective therapies.

Several studies have reported that an antigenic protein with a MW of approximately 30 kDa is present in *Blastocystis* organisms [17, 46] and differences in reactivity to this protein(s) between symptomatic patients and healthy controls have been reported [17, 47]. No reactivity with proteins of this size was reported in sera from patients with other parasitic infections suggesting the 30 kDa protein might be specific for *Blastocystis* organisms. A cytopathic monoclonal antibody, MAb1D5, previously shown to bind to the external surface of *Blastocystis* sp. [32], reacts with a30kDa protein from *Blastocystis* isolates derived from humans (ST’s unknown), but reportedly not with antigen derived from WR1 [24]. Peptide sequences derived from material obtained by 2-dimensional electrophoresis and Western blotting suggest that this 30 kDa protein is closely related to a cysteine protease of the legumain type. Although no reactivity of the MAb1D5 was reported previously with subtype WR1 [24],and this was the subtype we used as an antigen source in our study, the 27 kDa protein-reactivity that we observed more frequently in the symptomatic group may nevertheless correspond to this previously described 30 kDa protein. The difference in reactivity may be accounted for by increased purity of MAb1D5 used in our study and differences in antigen preparation technique, and the MW size difference accounted for by differences in *Blastocystis* antigen source and preparation (e.g., subtype, level of glycosylation). The MAb1D5 tested in parallel with our human serum samples reacted strongly with a double band around 100 kDa MW and also with a 27 kDa protein in the Western blots (Fig. 4). This pattern of reactivity (100 and ~30 kDa) against an antigen preparation using ST7 organisms was shown previously [27] and suggests that the 30 kDa antigen may be a processed or degraded form of the 100 kDa protein.

We also noted differences in IBS patients (IBS-P and IBS-N) with regard to reactivity to 50 and 75-95 kDa proteins. These proteins may correspond to the 50 and 118 kDa antigens [17], or the 60-100 kDa sized proteases [48] reported previously in symptomatic *Blastocystis* infected patients. Parasitic proteases are known to be highly immunogenic and may facilitate tissue invasion in the host. We searched for a protease that might be consistent with this role and sizes. Matrix metalloproteinase-9 (MMP-9) is one member of the zinc binding matrix metalloproteinases (MMP) that degrade components of the extracellular matrix including gelatin (gelatinase B), collagens, and elastin, thereby potentially aiding tissue invasion [49],and has been shown to interact with the immune system including potentiation of IL-8 [49]. MMP-9’s are found in the cytosol, vesicles and cell membranes of cells [50]. They exist as a pro-enzyme at around 92 kDa, an activated form around 84 kDa and truncated forms at lower sizes around 50 kDa in humans [51]. The pro-MMP-9’s are activated by other proteases, detergents and heat. We tested an antibody specific for human pro-MMP-9 in parallel with human sera in our Western blots and found that it reacted with *Blastocystis* proteins of approximately 75–100, 50 and 27 kDa MW, respectively, and that the pattern differed from the MAb1D5 reactivity. MAb1D5 reacted with a different distinct protein of approximately 95 kDa as well as a27kDa protein.

A subsequent zymogram performed using antigen that had been pre-treated with protease inhibitors minus EDTA showed a positive double band around 100 kDa that was abolished with EDTA. These findings suggest that a metalloproteinase around 100 kDa MW is present in *Blastocystis sp..* Inherent differences in gel runs and species variation of enzymes make it difficult to assess if this 100 kDa zymogram band is related to the Western blot 75–95 kDa bands we noted to be of significance in symptomatic patients. The findings nevertheless suggest that a MMP-9 isoform is present in *Blastocystis* organisms, and the pro-MMP-9 band may correspond to the 75–95 kDa protein, while the 50 kDa protein may possibly be the enzymatically active form and the 27 kDa form a truncated fragment.

The detection of a 27 kDa protein with both the anti-MMP-9 and the MAb1D5 suggest that this band may contain more than one protein, including a truncated fragment of a MMP-9 isoform. Previous studies investigating proteases present in *Blastocystis* sp. have suggested that cysteine proteases constitute the major class of proteases in this organism (80 % protease activity shown to be inhibited by cysteine protease inhibitors) [52]. However it may be difficult to account for proteases that are present as pro-enzymes that require activation or that may be inhibited from converting to active forms by other proteases. Protease activity at 32 kDa was reported to be significantly increased in symptomatic patients [47, 53] but may be due to a mixture of enzymes of that size. The genome of ST7 has been sequenced and this has allowed the prediction that all classes of proteases (cysteine, serine and metalloproteases) could be secreted by the parasite [26]. The majority (91 %) are predicted to be of the cysteine protease and none were specifically predicted, at this level of genome testing sensitivity, to be a metalloprotease of the MMP-9 class. Preliminary analysis of the ST4 genome has shown the presence of up to 30 % genes with no ortholog present in the ST7 genome [54] emphasising the genetic diversity present in this organism. Additionally, a recent study has discovered that 15 % of nuclear genes in *Blastocystis* sp. Subtype 7 uniquely use a polyadenylation-mediated creation of termination codons [55] and predicted genes may need to be re-evaluated in the light of this new information.

## Conclusions

Low serum IgA levels may facilitate *Blastocystis* carriage but are not associated with the development of IBS gastrointestinal symptoms. Antibodies to *Blastocystis* organisms are commonly found in the serum of many healthy individuals, but antibodies to 27, 50 and 75-95 kDa MW proteins are significantly increased in IBS patients regardless of *Blastocystis* status. Some of these serum antibodies are likely to be reacting to a previously reported cysteine protease, but others may be reacting with another class of proteases, the metalloproteases.

## Additional files

Additional file 1: Figure S1. Anti-proMMP-9, MAb1D5, MAb5 reacting with *Blastocystis* proteins in a Western blot. (PDF 292 kb) Additional file 2: Figure S2. Zymogram of *Blastocystis* WR1 proteins without addition of EDTA. (PDF 70 kb)

## Abbreviations

DNA: Deoxyribonucleic acid
EDTA: Ethylenediaminetetraacetic acid
GALT: Gut associated lymphoid tissue
HC: Healthy controls
HC-N: Healthy controls negative for Blastocystis spp.
HC-P: Healthy controls positive for Blastocystis spp.
IBS: Irritable bowel syndrome
IBS-N: Patients with irritable bowel syndrome negative for Blastocystis spp.
IBS-P: Patients with irritable bowel syndrome positive for Blastocystis spp.
IgA: Immunoglobulin A
IgG: Immunoglobulin G
IgM: Immunoglobulin M
IMDM: Iscove’s modified Dulbecco’s medium
kDa: kiloDalton
MAb: Monoclonal antibody
MMP: Promatrix metallopotease-9
MW: Molecular weight
PBS: Phosphate buffered saline
PBS/T: Phosphate buffered solution/Tween 20
PCR: Polymerase chain reaction
SDS-PAGE: Sodium dodecyl sulphate polyacrylamide gel electrophoresis
ST: Subtype
S: Subunit
Sp.: Species
rDNA: Gene coding for ribosomal ribonucleic acid
XIVC: Xenic *in vitro* culture
