# Supplementary material for: Blastocystis specific serum immunoglobulin in patients with irritable bowel syndrome (IBS) versus healthy controls
Source: Parasit Vectors. 2015 Sep 15;8:453. doi: 10.1186/s13071-015-1069-x (PMC4572630; doi:10.1186/s13071-015-1069-x)
Supplement: Additional file 1: Figure S1. — Anti-proMMP-9, MAb1D5, MAb5 reacting with Blastocystis proteins in a Western blot. (PDF 292 kb) [file 13071_2015_1069_MOESM1_ESM.pdf]

Supplementary Figure: S1
Anti-proMMP-9, MAb1D5, MAb5 reacting with *Blastocystis* proteins in a Western blot*

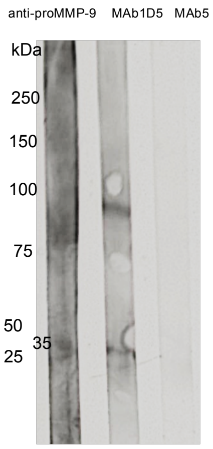

anti-proMMP-9=antibody to pro-matrix metalloprotease-9 Lane 1
MAb=monoclonal antibody MAb1D5 (Lane 2) and MAb5 (Lane 3)
kDa=kiloDalton molecular weight
*7.5% gel
