# Supplementary material for: Blastocystis specific serum immunoglobulin in patients with irritable bowel syndrome (IBS) versus healthy controls
Source: Parasit Vectors. 2015 Sep 15;8:453. doi: 10.1186/s13071-015-1069-x (PMC4572630; doi:10.1186/s13071-015-1069-x)
Supplement: Additional file 2: Figure S2. — Zymogram of Blastocystis WR1 proteins without addition of EDTA. (PDF 70 kb) [file 13071_2015_1069_MOESM2_ESM.pdf]

Supplementary File: S2

Zymogram of *Blastocystis* WR1 proteins without addition of EDTA

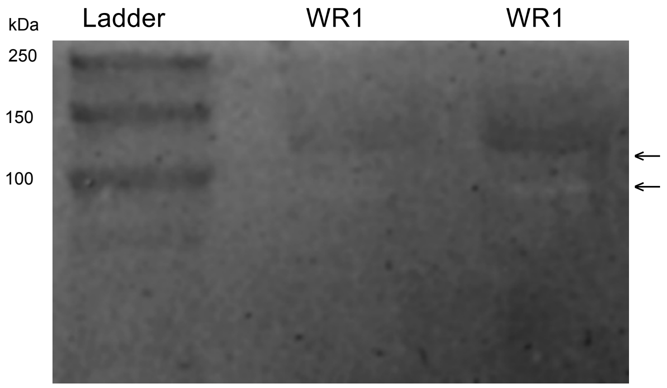

Arrows indicate level of positive zymogram bands
